# Genetic Structure Analysis of the Pura Raza Español Horse Population through Partial Inbreeding Coefficient Estimation

**DOI:** 10.3390/ani10081360

**Published:** 2020-08-06

**Authors:** Davinia I. Perdomo-González, María J. Sánchez-Guerrero, Antonio Molina, Mercedes Valera

**Affiliations:** 1 Departamento de Ciencias Agro-forestales, ETSIA, Universidad de Sevilla, Carretera de Utrera Km 1, 41013 Sevilla, Spain; v32sagum1@gmail.com (M.J.S.-G.); mvalera@us.es (M.V.); 2 Departamento de Genética, Universidad de Córdoba, Campus Universitario de Rabanales, Edificio Gregor J. Mendel, Planta baja, Carretera Madrid-Cádiz km 396ª, 14071 Córdoba, Spain; ge1moala@uco.es

**Keywords:** common ancestors, genetic variability, studbook, genealogical analysis

## Abstract

**Simple Summary:**

The Pura Raza Español horse (PRE) is an autochthonous Spanish horse recognized as an official breed since the 15th century. In 1912, with the creation of its studbook, it became a closed population (only animals belonging to the breed acts as breeders: 23,530 stallions and 75,870 mares), in which relatedness between individuals and inbreeding has tended to increase. Inbreeding estimation measures the probability in an individual of possessing, in one gene, two identical alleles derived from a common ancestor. Each common ancestor provides part of the descendant’s total inbreeding (partial inbreeding coefficient, F_ij_). This work analyzes the F_ij_, calculated using a recently developed approach (based on founders and Mendelian sampling of the non-founders) in the whole PRE population. The identification of 10,244 common ancestors and their relationship with the seven most influential individuals of the breed allowed us to determine that a genetic bottleneck due to an unequal contribution over the generations occurred. Computing F_ij_ has being an important tool to determine which are the breeding animals, with more or less massive use, whose are determining a loss of genetic variability in the population at each moment and has enabled us to expand our knowledge about PRE genetic demographic evolution.

**Abstract:**

The aim of this work was to analyze genetic parameters such as the inbreeding coefficient (F), relatedness coefficient (AR) and partial inbreeding coefficient (F_ij_) of the whole PRE population, and the ancestors which account for 50% of the total genetic variability of the current population, from genealogical information. The average F of the whole PRE population (328,706 animals) has decreased from 8.45% to 7.51% in the least 20 years. The F_ij_ was estimated for the whole PRE population, resulting in a database of 58,772,533 records containing one record for each F_ij_ that each animal receives from a certain common ancestor (CA). A total of 10,244 CAs contributed to the F_ij_ with an average of 5370 descendants, with each descendant having an average of 170 CAs. Over the generations, the number of CAs has increased, while the proportion of F_ij_ by each one has decreased. In addition, the contributions of the more influential ancestors have changed. The increased census, the limited use of artificial insemination and our increased knowledge about inbreeding depression and the animals’ breeding values allow breeders to select horses more for their functionality and conformation than for their pedigree reputation, which is the cause of all these changes.

## 1. Introduction

The Pura Raza Español horse (PRE) is an autochthonous Spanish horse population and the most important horse breed in Spain in terms of both history and census, with 252,852 active horses [1]. A single association, the National Association of Pure Spanish Horse Breeders (ANCCE), manages all of them, despite the fact that 23.3% of PRE horses are distributed abroad, in over sixty countries [1]. PRE is a breed with ancestors dating back thousands of years in the Iberian Peninsula, and was recognized as an official breed in the 15th century [2]. This breed has exceptional temperamental and functional qualities, making it highly suitable for different equine competitions, above all, classic dressage [3]. The foundation of the studbook was officially approved on January 1, 1912, and it was being managed by the Spanish Armed Forces first and since 2007 by the ANCCE. Following the creation of the studbook, the number of horses born each year increased (around 100 animals per year) until the 1970s, after which the growth surged to a maximum figure of 19,560 new horses in 2009. From that year, growth has fallen and has now stabilized at about 10,000 births per year. Since the establishment of the studbook, the inscription of new PRE horses has been restricted to those animals born from horses registered in the official studbook, which has therefore created a completely closed studbook, in which the mating of related individuals is inevitable in the long term. In closed populations with a non-reduced census, the intensive use of selection also leads to an increase in the level of inbreeding [4]. Pedigree-based relationship coefficients are often used to manage the inbreeding in a population when effects of the inbreeding depression begin to be manifested. Although, in recent decades, attempts have been made to maintain genetic variability in the PRE population, in general, unbalanced contributions of founders reflect the great loss of genetic diversity over the generations [2].

The inbreeding coefficient of an individual (F) is defined as the probability of an individual to possesses two identical alleles by descent from a common ancestor at the same randomly chosen locus [5]. Historically, F has been studied only as the sum of the founder’s partial coefficients [6], but this does not provide detailed information about the contribution of each ancestor to the identity-by-descent (IBD) probability. Alternatively, F can be broken down into the sources of the coancestry between the parents of each individual [7,8] as the sum of the partial coefficient from founders and the Mendelian sampling of the non-founders. These partial coefficients into which F can be partitioned are known as partial inbreeding coefficients, F_ij_, which is the combined probability that an individual *i* is autozygous for an allele and that this allele was derived from the allele in ancestor j [6,9,10].

The possibility of computing, for the first time in equines with a high census population, the partial inbreeding coefficients (F_ij_) attributed not only to founders but also to non-founders, presents a way of analyzing the PRE population structure and evolution from a new point of view and gives us greater insight into the genetic variability of PRE over the generations. Moreover, this approach provides us with a methodology to identify the unequal common ancestor’s contribution and determine the reasons why a certain ancestor becomes important.

## 2. Materials and Methods

Pedigree data were obtained from the ANCCE studbook, with a total of 328,706 animals (160,640 males and 168,066 females) born from 1900 to 2018, of which 99,400 horses (30.24%) have been used for breeding (23,530 stallions and 75,870 mares).

The parameters related to the quality of genealogical information were estimated, including *pedigree completeness level* (the proportion of ancestors known in each ascending generation [11]), *complete generations* (the number of generations separating the offspring of the furthest generation where the 2^g^ ancestors of the individual are known, where *g* is the number of generations since the last known ancestor), *maximum generations* (the number of generations separating the individual from its furthest ancestor) and *equivalent generations* (the sum of (1/2)n, where n is the number of generations separating the individual to each known ancestor [12]). The parameters of genetic variability estimated were *inbreeding coefficient* (F, defined as the probability that an individual has two genes identical by descent [13]) and *average relatedness coefficient* (AR, defined as the probability that an allele randomly chosen from the whole population in the pedigree belongs to a given animal [14], i.e., the representation of the animal in the whole pedigree; also, the AR of a founder indicates its genetic contribution to the population). Finally, the *number of founders* (ancestors with unknown parents), the *number of ancestors* (the ancestors, not necessarily founders, accounting for 100% of the genetic diversity in the actual population), the *effective number of founders* (f_e_, the number of equally contributing founders that would be expected to produce the same genetic diversity as in the population under study [15]) and the *effective number of ancestors* (f_a_, the minimum number of ancestors, not necessarily founders, which account for the complete genetic diversity of a population [15]) were also calculated to evaluate whether bottlenecks have affected the population.

The approach developed by Casellas [16] was used to estimate the F_ij_ transmitted by each common ancestor to each inbred descendant of the PRE pedigree file. This new approach allowed the inbreeding to be broken down into the sources of the co-ancestry between the parents of each individual, which include the founders of the population (the traditional approach) and the Mendelian sampling of the non-founders [7,8]. Thus, the inbreeding load (*i_i_*) for the *i* th individual can be decomposed as follows:

(1) $$i_{i}=i_{s}+i_{d}+\varepsilon_{i}$$

where *i_s_* and *i_d_* are the inbreeding loads for its sire and dam, respectively, and $\varepsilon_{i}$ is its Mendelian sampling [16,17]. This approach provides a subset of partial inbreeding coefficients accounting for the identity-by-descent contribution from each relevant ancestor. In addition, to evaluate whether the most influential ancestors (those which provide the F_ij_ for a greater number of descendants) also explain most of the genetic variability of the total PRE population, ancestors accounting for 50% of the genetic contribution in the total population (based on Boichard methodology [15]) were analyzed. Demographic and genetic variability parameters were calculated using the ENDOG v4.8 software [18].

Finally, with the objective of identifying which factors have contributed to a certain animal having a higher or lower inbreeding level, several factors historically related to F were analyzed using a general linear model followed by a Tukey post hoc test of least square means. Reproductive status (4 levels; male without offspring, female without offspring, stallion with offspring and mare with offspring) was included to determine if linage implies a higher or lower use of an animal for reproduction. Coat color (5 levels; grey, bay, black, chestnut and other minority coat colors) was included to analyze its pleiotropic role. And factors as birth generational interval (10 levels; 1919–1928, 1929–1938, 1939–1948, 1949–1958, 1959–1968, 1969–1978, 1979–1988, 1989–1998, 1999–2008 and 2009–2018), birth stud size (7 levels; 1 animal, 2–4 animals, 5–9 animals, 10–14 animals, 15–19 animals, 20–24 animals and ≥25 animals) and geographic zone (6 levels; Spain, Rest of Europe, South America, North America, Australia and Africa) were included due to its relation to differences in horse management. Statistical analyses were performed using SPSS software for windows [19].

## 3. Results

The pedigree completeness level of the PRE horses born in last generational interval (the last 10 years [20], 115,005 individuals) was 99.6%, remaining above 99% until the third generational interval and above 90% until the seventh generational interval. The mean complete generations of the total pedigree (328,706 horses) were 5.6, the mean maximum generations were 17.1 and the mean equivalent generations were 9.4. The total PRE population showed an average F of 7.51% and a mean AR of 11.31%. Up to 98.7% of the registered individuals are inbred, and those born in the last four generations are 99.9% inbred. Animals belonging to the last generational interval showed a mean F of 7.25% and a mean AR of 11.20%, with the mean of complete generations 6.2, a maximum pedigree depth of 18.8 generations and the mean of equivalent generations of 10.4. The effective number of founders (f_e_ = 33) was only 3.13% of the total number of founders (1053) and the effective number of ancestors (f_a_ = 19) was 1.86% of the total number of ancestors (1023).

The F_ij_ was estimated for the whole PRE population, resulting in a database of 58,772,533 records (one record for each F_ij_ that each animal receives from a certain common ancestor). The F_ij_ values oscillated from 0% to 25% and the average F_ij_ (±sd) was 0.042% (±0.19). A total of 10,244 common ancestors (3676 stallions and 6568 mares) provided an F_ij_ for an average of 5370 offspring (from 1 to 323,807 descendants). The number of common ancestors for each descendant ranged from 1 to 731 (an average of 170). Most of the 58,772,533 F_ij_ records were very low; 92.0% were lower than 0.1%. The remaining 8% were F_ij_ values equal to or above 0.1% belonging to a total of 323,803 animals, which received F_ij_ from at least one common ancestor and a maximum of 49 (an average of 15 CAs). A total of 9778 CAs (3569 stallions and 6209 mares) provided an F_ij_ ≥ 0.1% for an average of 487 descendants (from 1 to 317,790).

The total PRE population was divided according to different groups of F values showing the average number of common ancestors (ACA) and the average F_ij_ they transmit in each group (Table 1). Almost half the PRE population (47.45%) have an F value between 3.12% and 6.25% and, at the same time, this population group has the highest number of male common ancestors (an average of 84). The F ≥ 25% population group has the same average number of male common ancestors (an average of 84) but the highest number of female ancestors (an average of 108). The least numerous groups are the extremes: 0% < F < 3.125% and F > 25.0% with 5.03% and 1.29% of the population, respectively. Besides, animals with a higher F value (≥25%) also have common ancestors which transmit a higher average F_ij_, both in stallions and mares. In all cases, males transmitted a higher average F_ij_ value (from 0.04% to 0.41%) than females (from 0.02% to 0.19%).

**Figure 1:**
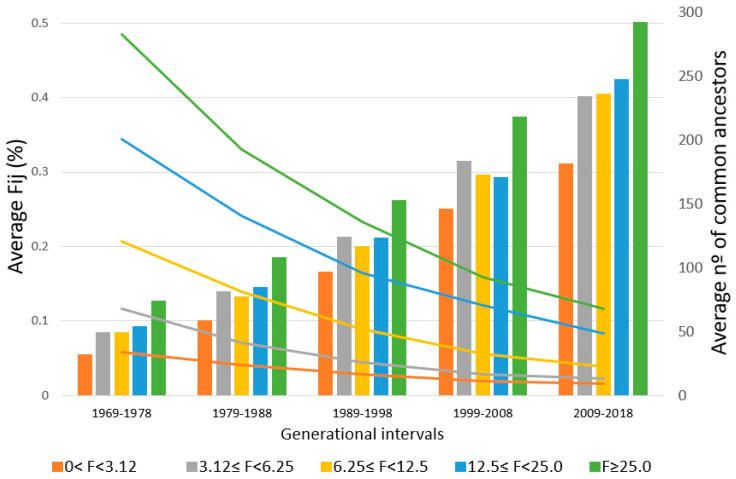
Average partial inbreeding coefficient (F_ij_) by common ancestor (lines) and average number of common ancestors (bars) in the last 5 generational intervals according to the total inbreeding coefficient (F) level of the Pura Raza Español horse.

On the other hand, the average partial inbreeding coefficient transmitted by common ancestors and the ACA in individuals (320,483 horses) born in the last five generational intervals are shown for each F group (Figure 1). It can be seen that the ACA (bars), by a group of F values, is increasing each generational interval, while, at the same time, the average F_ij_ transmitted by common ancestors (lines) decreases. In the 1969–1978 interval, horses with an F coefficient higher than 0% but lower than 3.12% have an ACA of 32, transmitting an average F_ij_ of 0.06%, while horses with an F coefficient ≥25% have an ACA of 74, transmitting an average F_ij_ of 0.49%. On the other hand, in the 2009–2018 interval, inbred horses with an F coefficient lower than 3.12% have an ACA of 182, with an average F_ij_ of 0.02%, while horses with an F coefficient ≥25% have an ACA of 293, with an average F_ij_ of 0.12%.

**Figure 2:**
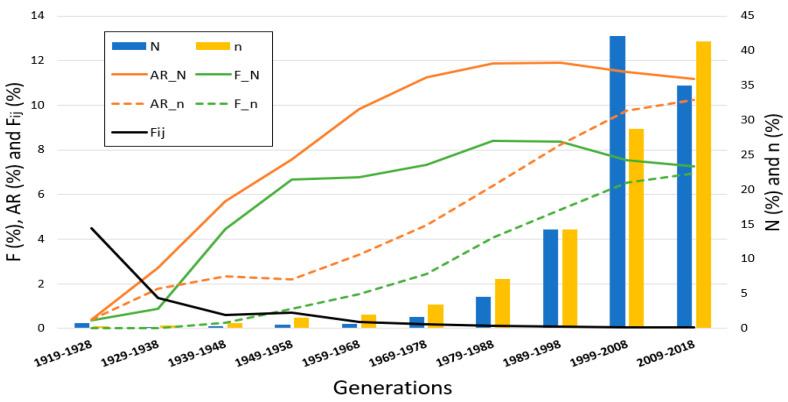
Census evolution of the Pura Raza Español horse (N = 328,706) and their common ancestors (n = 10,244), average inbreeding coefficient (F_N and F_n), average relatedness coefficient (AR_N and AR_n) and average partial inbreeding value (F_ij_) by generational intervals.

The PRE population evolution (N) by generation (Figure 2), highlighting a major increase in horses born over the last two generations (1999–2018), in which 253,525 horses were born, more than the 77% of the whole PRE population. Over the generations, the average of common ancestors (n) by generation of birth has also increased. The average F value for the PRE population (F_N) increases from 0.36% in the first generation to 8.40% in the 1979–1988 interval and then decreases to 7.25%, while the average F of their common ancestors (F_n) increases gradually from 0% to 6.96%. The average AR behaved in a similar way, with each generation increasing from 0.38% to 11.91% until 1989–1998 for the total population (AR_N) with a minor decrease over the last two generations to 11.19%. The average AR of their common ancestors (AR_n) rose slowly from 0.40% to 2.21% in the first four generational intervals (from 1919 to 1958) and then increased more sharply to 10.25% in the last generation interval. Finally, the average F_ij_ that the common ancestors transmitted to their descendants has seen a gradual decrease over the generations, from 4.48% to 0.03%.

On the other hand, the total genetic variability of the PRE population can be explained by just 1023 ancestors (f_a_, founders or not), with 50% of the total contribution due to only seven of them, five males and two females (Table 2). These seven most influential ancestors, born between 1900 and 1949, transmit an average F_ij_ ranging from 0.03% to 0.54% to an average of 288,644 descendants (between 182,731 and 319,917). Despite this large number of descendants, very few of them have received an F_ij_ equal or higher than 6.25% from these 7 ancestors, including only 33 descendants from *Maluso*, 31 from *Americano*, 30 from *Celoso III*, 24 from *Destinada*, 5 from *Oficial I* and 0 from *Oficial III* and *Zurrona*. Between 30% and 40% of the descendants of these seven ancestors are in the last generational interval (except *Maluso*, with only 4.92%), transmitting them an average F_ij_ slightly lower than that of their total descendants. The contributions of the two major ancestors, *Americano* and *Destinada*, are 13.9% and 11.8%, respectively, which represents over 25% of the total genetic contribution. Both *Americano* and *Destinada* have slowly increased their influence over the generations, with a maximum influence in 1999–2008, when they had the highest number of descendants (43.7% and 43.3% of their total descendants, respectively). After that, their number of descendants decreased to 36.34% and 35.94%, respectively, in 2009–2018 (with around 80% of their descendants born in the last two generational intervals). The F_ij_ transmitted by *Americano* and *Destinada* have followed the same tendency as the average, decreasing from 6.25% and 3.75% in the 1919–1928 interval to 0.45% and 0.67% in the 2009–2018 interval, respectively, so *Americano* at first transmitted a higher F_ij_ and currently transmits a lower F_ij_ than *Destinada*. Moreover, these seven most influential ancestors (based on Boichard methodology [15]) are part of the 5% (520 common ancestors) of the total ancestors (based on Casellas methodology [16]) with the largest number of descendants, occupying positions 15, 16, 19, 38, 40, 45 and 153. These 520 ancestors transmit an average F_ij_ of 0.03% to an average of 108,960 descendants.

Finally, to identify which factors, previously related to F, could have contributed to a horse having a higher or lower inbreeding level, a general lineal model was used. All the factors studied were significantly associated with the F level (Table 3). The results related with reproductive status showed that breeding horses (both stallions and mares with offspring) have a higher mean F than those without offspring; furthermore, stallions show an average F higher than mares. In addition, horses with grey coats, the birth generational interval between 1979 and 1988, a birth stud with more than 25 horses and horses from South America showed the highest mean F values.

## 4. Discussion

As the inscription of new PREs since 1912 has been restricted to the offspring of horses already registered, the mean F of the population increased until the 1990s [21], followed by a decrease in the last two decades. The mean F (7.51%) and AR (11.31%) values obtained in this work were lower than the values reported in the same breed by Valera et al. [21], F = 8.48% and AR = 12.25%, Cervantes et al. [22] F = 8.20% and Gomez et al. [23] F = 8.20%. This is mainly because these authors did not include data from the two last decades, when over 70% of the PRE population was born and when management by breeders and the breeding program have become a key part of conservation genetics, aimed at avoiding matings with high levels of inbreeding [24].

Several studies have reported different average inbreeding values, which are positively correlated to pedigree length and completeness [21]. The greater the depth of the pedigree, the greater the possibility of have a greater number of common ancestors (in pedigrees with a high depth and high completeness, the increase in its depth has little impact on his F). This can be observed in other closed populations, such as the Spanish Arabian horse, with an F value of 7% and a mean equivalent generation of 7.9 [25], or the Old Kladruber horse, with an F value of 13% and an equivalent generation number of 15.1 [26]. The Lusitano and Lipizzan horse breeds are historically and genetically related to the PRE breed [27,28]. The Lusitano horse breed shows a mean F value of 9.92% and a number of equivalent generations of 9.87 [27], while the Lipizzan horse, with 15.22 complete equivalent generations, shows a mean F of 10.8% [28]. In addition, despite the fact that the Lusitano is a closed and inbred breed, the care taken by breeders to maintain the genetic variability, as the case of the Lusitano horse in Brazil, make their level of inbreeding lower than the total population, especially in the last generation with a mean F of 4.46% and a number of equivalent generations of 6.4 [29].

In order to determine the main factors that define a greater or lower level of inbreeding in the current PRE population, a number of factors, such as coat color, reproductive status, birth generational interval, geographic zone and birth stud size have been evaluated. All the factors studied are associated with the F level in our population with a statistically high significance. Coat color has been previously determined as a highly significant factor in other analyses of the PRE breed, which studied reproductive parameters [20], morphology [30] and defects such as vitiligo and melanoma [31] or cresty neck [32], highlighting an association with the pleiotropic role of coat color genes, especially with the grey coat color. The reason why the grey coat color is especially related to high levels of inbreeding could be attributed to the influence of the Carthusian strain to PRE horses. This strain is highly inbreeding, and its influence in the PRE population is very raised [21]. However, the mating of certain animals for their coat color is more related to a trend of the moment than an inbreeding issue, because, throughout history, certain coat colors have been more desirable than others [20]. Reproductive status seems to be an important factor in this breed, where the individuals with important ancestors continue to be used as breeder animals due to their desirable racial traits, established by breeders over the years through inbreeding, meaning that the most inbred horses are used as breeding animals. In addition, the reproductive status of the mare was used as a factor related to consanguinity in the fertility of Black Forest Draught horses [33], where a major influence on the cycle foaling rate was found. Meanwhile, the generational interval seems to be a factor that contributes significantly to our knowledge of mean F tendencies. Inbreeding tendencies in a breed history tend to increase gradually, as occurs in the Maremmano horse breed [34] with an increase from 1.29% (1981) to a maximum of 5.38% (2015), or can remain constant, as in the Lusitano breed, which initially showed a stable average F value pattern of around 10% until the 1980s and then increased slightly [27]; in other cases, it can decrease, as in the PRE, whose F started to decrease in the 1990s. These differences in inbreeding tendencies depend on both the effective population size and whether breeders have been working to stop the loss of variability or not. Finally, the geographic zone has been marked as a factor of low relevance [31,32]; nevertheless, it has been studied due to its relation to differences in horse management, such as the birth stud size factor, where the highest mean F values are produced in those countries and studs with largest horse census. This can be attributed to, on the one hand, the scarce genetic exchange between geographic zones and, on the other, the intensive or non-intensive use of certain breeders depending on the stud size.

The fact that f_e_ (33) is much lower than the real number of founders (1053) indicates a big loss of genetic variability. In the same way, f_a_ (19) is much lower than the number of ancestors which account for 100% of the genetic variability (1023), which shows that this loss of genetic variability from the parents to their progeny was the result of a genetic bottleneck. The results of the present study show that f_e_ was lower, whereas f_a_ was higher than the values obtained by Valera et al. [21], 39 and 16, respectively, two generational intervals ago, demonstrating a lessening of the genetic erosion over the last few years, when inbreeding has been actively avoided by breeders. Genetic variability lost due to a bottleneck can be predicted using 1/(2f_a_) and the current figure is 2.63%, a lower value than two generational intervals ago (3.03%). The f_e_/f_a_ ratio found in this study was 1.74, which indicates a decrease in genetic variability caused by an unequal founder contribution, i.e., some breeding animals become popular and produce more progeny than others. This has also been seen in other horse breeds, such as the Campolina horse population [35] with an f_e_/f_a_ ratio of 1.51, the Spanish Arab Horse [25] with a ratio of 2.03% or in the Old Kladruber horse with a ratio of 5.40 [26].

Some studies revealed that closed populations, with multiple generations of intense directional selection [36] and high reproductive success due to the use of advanced reproductive technologies [37,38], tend to accumulate inbreeding and lose genetic variability. In our case, there is growing awareness about the loss of genetic variability, therefore, breeders make a restrained use of reproductive technologies to pair animals from different studs. This has led to a stabilization and a decrease in PRE inbreeding over the last three decades. At the same time, having a ranking of all the animals with positive evaluations for a variety of selection traits, such as dressage [3] or conformation [39], has allowed breeders to use stallions and mares that stand out more for their genetic evaluation and less for their relationship with their common ancestors. Even when all this is taken into account, some of the most important PRE studs have serious problems due the high level of F and the consequent inbreeding depression, which is clearly evidenced in reductions of body measurements in this breed [23].

Regarding the number of common ancestors by the group of F, it is notable that the F group with the most individuals (F between 3.12% and 6.25%) has a similar ACA to the least numerous group, F group ≥25.0%, both for stallions (84 and 84, respectively) and for mares (97 and 108, respectively). The main difference is the proportion of F_ij_ that the CA transmit to their descendants, which is much higher in the second group (0.41% by stallions and 0.19% by mares) than in the first (0.04% by stallions and 0.02% by mares). The main consequence of this is that knowing the amount of F_ij_ that each CA contributes to a descendant is more relevant than knowing its total number of ancestors. Moreover, the fact that horses with F ≥ 25% have common ancestors that transmit the highest average F_ij_ (both stallions and mares) concurs with the idea that the mating of highly inbred common ancestors results in offspring which also show high F values. In addition, despite the fact that stallions transmit the highest F_ij_ to their descendants, the number of mares that become common ancestors is higher.

The first researchers to work with the concept of F_ij_ were James and McBride [40], who traced the percentages of genes contributed by founders in a closed poultry flock from pedigree information. Nowadays, although different publicly available programs exist for calculating F_ij_, none of them was able to combine the partial inbreeding coefficient from founders and the Mendelian sampling of non-founders until the appearance of the Casellas [16] approach. These authors worked with a Maret rabbit population (2657 animals), in which a total of 43,635 F_ij_ values were obtained, ranging from 0.002% to 28.1%, and they described that each inbred rabbit had between 1 and 42 CAs. At a later stage, Varona et al. [17] worked with beef cattle data, in the Pirenaica (308,836 animals) and Rubia Gallega (384,434 animals) breeds. Ours is the first work to analyze equine breed data with this methodology. Pirenaica and Rubia Gallega beef cattle data generated more than 16 and 5 million F_ij_ from 8721 and 3601 common ancestors and an average F_ij_ of 0.15% and 0.057% with 5.9 and 3.9 average generations, respectively. These results imply that the PRE population generates the biggest amount of F_ij_ values from a higher number of common ancestors (10,244), a lower average F_ij_ value (0.042%) and an intermediate census (328,706 animals) and complete generations (5.6). This means that the fact that the PRE average F value is considerably higher than that of Pirenaica (3%) and Rubia Gallega (2%) is due to the large number of ancestors that transmit a low F_ij_ rather than to a low number of ancestors transmitting a high F_ij_. This fact can be observed in Figure 1, too, where animals with the same F levels have a greater number of common ancestors with a lower average F_ij_ when the current generation is compared with five generations ago.

As stated above, the last two generational intervals represent over 77% of the individuals registered in the PRE studbook. From the studbook’s creation, in 1912, there has not been any change in horse or foal registration policies, just the intensive efforts made by breeders to improve the breed and make it more international have allowed this great increase, which shows that this breed is experiencing a period of rapid growth. In addition, the efforts made in the breeding program to decrease inbreeding (with selection and mating processes) have resulted in an increased number of animals becoming common ancestors, which has already grown exponentially each generation. Inbreeding and average relatedness values have shown similar behavior over generational intervals in both the total PRE population and its common ancestors. It seems that, in the last generation, the increase in the average AR values was less marked, which demonstrates that breeders are looking for less closely related individuals and avoiding inbreeding, due to the fact that using animals with low AR values as breeders maintains the genetic variability. On the other hand, over the generations, while the number of common ancestors has increased, the average F_ij_ that a common ancestor transmits to its descendants has decreased, the opposite of which would be expected since the PRE breeding program started to be implemented in 2003. The PRE breeding program is managed by ANCCE, and includes functional conformation, dressage and riding abilities, in addition to morphology, as the main selection criteria [41,42]. The fact the average F_ij_ that a common ancestor transmits to its descendants has decreased is mainly due to the increasing awareness among horse breeders about the inbreeding depression [2] and the better information available about the animals’ breeding values, which enables them to choose the best stallions and mares (also with the lowest AR) to obtain foals with the desired characteristics and less F, thus reducing the average founder’s contribution to the population [34].

Nowadays, despite the large Pura Raza Español horse census, just 1023 ancestors account for 100% of the genetic variability, only seven of which are responsible for 50% of the population gene pool, which is one more than reported by Valera et al. [21]. *Americano* and *Destinada* remain the two ancestors with the highest contribution percentages, despite making a lower contribution to the total gene pool than two generational intervals ago (*Americano* contribution has decreased from 15.7% to 13.9% and that of *Destinada* from 12.6% to 11.8%). This coincides with the fact that both ancestors had a lower percentage of descendants in the last generation than one generation before, which means that their influence increased exponentially until the 1999–2008 interval but has decreased in the last generation. On the other hand, *Maluso* now accounts for a bigger contribution percentage (from 5.37% to 6.7%) and *Celoso III* (from 6.27% to 6.0%) and *Zurrona* (from 3.62% to 3.1%) for smaller percentages. As regards the F_ij_ they transmit, *Americano* and *Destinada* transmit to their descendants the highest average F_ij_ values, 0.54% and 0.74%, respectively while *Maluso*, with an average transmitted F_ij_ of 0.24%, transmits a large proportion of partial inbreeding (≥6.25%) to the highest number of descendants, followed by *Americano* and *Celoso III*. This fact could explain why *Maluso* has the lowest number of descendants in the last generation (only 5% of his total descendants), while the other ancestors have around 40%, probably because of the visible effects of inbreeding depression as reduction in reproductive parameters or morphology measurements.

## 5. Conclusions

Breeders design horse’s matings in function of their breeding objectives, looking to fix certain characteristics and the maximum influence of animals with great value, genetic or phenotypic. The general idea in the past that mating related individuals establishes certain long-term traits is demonstrated here with the use of certain animals from both the maternal and paternal lineages, making them common ancestors.

To achieve this, apart from controlling inbreeding, the assessment of genetic contributions from founders and ancestors of the current population has provided us with a better understanding of the changes that take place in the genetic pool of this breed. The efforts made to reduce the average F over the last generations have been mainly possible thanks to our increased knowledge about the animals’ relatedness (offspring’s inbreeding) and to the effectiveness of the breeding program to provide breeders with breeding values, which increases their options of successfully choosing new animals regardless of their pedigree or the breeder’s prestige. Therefore, in addition to carrying out a systematic control of the inbreeding level both in the total population and within studs, it is important to determine the reasons that a certain ancestor is being used so intensely that it determines an increase in the kinship between the breeding animals and therefore a loss of variability, both in the total population and within certain studs.

## Figures and Tables

**Table 1:** Partial inbreeding coefficient (F_ij_) transmitted by common ancestors classified by the total inbreeding coefficient (F) level of the Pura Raza Español horse.

F Group (%)	N	N (%)	Average n° of Common Ancestors [Min, Max]		Average F_ij_ (%) ± SD	
			Stallions	Mares	Stallions	Mares
>0 to 3.12	16,518	5.03	51 [1, 142]	55 [1, 181]	0.04 ± 0.07	0.03 ± 0.07
3.12 to 6.25	155,984	47.45	84 [1, 209]	97 [1, 315]	0.04 ± 0.05	0.02 ± 0.04
6.25 to 12.5	109,641	33.36	76 [1, 233]	89 [1, 353]	0.09 ± 0.13	0.05 ± 0.08
12.5 to 25.0	38,008	11.56	72 [1, 283]	89 [1, 448]	0.21 ± 0.37	0.11 ± 0.24
≥25.0	4242	1.29	84 [1, 225]	108 [1, 355]	0.41 ± 1.2	0.19 ± 0.31

**Table 2:** Ancestors accounting for 50% of the genetic variability of the total Pura Raza Español horse (PRE) population. The table shows their contribution, the number of total descendants, those with an F_ij_ value over 6.25%, the number and percentage of descendants in last generational interval, total average F_ij_ and the average F_ij_ they transmit to the last generational interval.

Ancestors	Sex	Year	C(Ca)	N(1)	F_ij_ (%)	N(2)	N(3)	% N(3)	F_ij_(%)_N(3)_
Americano	M	1927	13.9	316,293	0.54	31	114,952	36.34	0.45
Destinada	F	1923	11.8 (25.7)	319,917	0.74	24	114,981	35.94	0.67
Maluso	M	1949	6.7 (32.4)	182,731	0.24	33	89,887	4.92	0.20
Celoso III	M	1933	6.0 (38.4)	296,641	0.11	30	113,587	38.29	0.09
Oficial III	M	1927	4.9 (43.3)	295,296	0.07	0	113,408	38.40	0.06
Oficial I	M	1900	4.2 (47.5)	317,585	0.09	5	114,981	36.20	0.09
Zurrona	F	1919	3.1 (50.6)	292,045	0.03	0	112,552	38.54	0.02

Year: ancestors birth’s year; C: contribution (%); Ca: accumulated contribution (%); N(1): ancestors’ total number of descendants; F_ij_ (%): average F_ij_ transmitted; N(2): ancestors’ descendants with F_ij_ ≥ 6.25%; N(3): number of descendants born in last generation; %N(3): Percentage of descendants born in last generation; F_ij_(%)_N(3)_: average F_ij_ transmitted to descendants born in last generation.

**Table 3:** Main factors affecting inbreeding coefficient (F, %) in the total population of Pura Raza Español horses. Means comparison with Tukey Least Square Means post hoc test.

n/F(%)	Effect/Levels										*p* Value
	**Reproductive Status**										
	Male	Female	Stallion	Mare							
n	137,110	92,196	23,530	75,870							
F (%)	7.50 ^ab^	7.45 ^a^	7.74 ^c^	7.55 ^b^							<0.001
	**Coat Color**										
	Grey	Bay	Black	Chestnut	Others						
n	147,885	92,815	29,022	8324	647						
F (%)	8.19 ^b^	6.80 ^a^	6.84 ^a^	6.93 ^a^	7.83 ^b^						<0.001
	**Generational Interval**										
	1919–1928	1929–1938	1939–1948	1949–1958	1959–1968	1969–1978	1979–1988	1989–1998	1999–2008	2009–2018	
n	2406	659	972	1756	2174	5323	15,127	46,764	138,520	115,005	
F (%)	0.36 ^a^	0.87 ^a^	4.44 ^b^	6.66 ^c^	6.78 ^c^	7.33 ^d^	8.40 ^f^	8.37 ^f^	7.54 ^e^	7.26 ^d^	<0.001
	**Birth Stud Size (Number of Horses)**										
	1	2–4	5–9	10–14	15–19	20–24	≥25				
n	4515	15,181	19,470	15,027	11,615	10,280	159,683				
F (%)	6.57 ^a^	6.50 ^a^	6.63 ^a^	6.81 ^b^	7.00 ^c^	7.12 ^c^	7.75 ^d^				<0.001
	**Geographic Zone**										
	Spain	Rest of Europe	South America	North America	Australia	Africa					
n	231,105	15,334	23,094	11,576	856	203					
F (%)	7.64 ^a^	7.13 ^b^	8.14 ^c^	7.52 ^a^	7.63 ^a^	6.73 ^ab^					<0.001

n: number of horses; F: inbreeding coefficient; ^a–f^ Different superscript letters indicate significant difference between levels.
